# The Influence of Berberine on Vascular Function Parameters, Among Them VEGF, in Individuals with MAFLD: A Double-Blind, Randomized, Placebo-Controlled Trial

**DOI:** 10.3390/nu17223585

**Published:** 2025-11-16

**Authors:** Anna Koperska, Ewa Miller-Kasprzak, Agnieszka Seraszek-Jaros, Katarzyna Musialik, Paweł Bogdański, Monika Szulińska

**Affiliations:** 1 Department of Treatment of Obesity, Metabolic Disorders and Clinical Dietetics, Poznan University of Medical Sciences, 60-355 Poznan, Poland; 2 Doctoral School, Poznan University of Medical Sciences, 60-812 Poznan, Poland; 3 Department of Bioinformatics and Computational Biology, Poznan University of Medical Sciences, 60-529 Poznan, Poland

**Keywords:** berberine, BBR, MAFLD, MASLD, NAFLD, VEGF, PWA, endothelium

## Abstract

Background: Metabolically Associated Fatty Liver Disease (MAFLD) is a prevalent liver disorder closely tied to metabolic dysfunction, insulin resistance, and chronic low-grade inflammation. Vascular Endothelial Growth Factor (VEGF) may have a dual interesting role in MAFLD pathophysiology—supporting vascular repair in early stages, but potentially contributing to fibrosis in later stages. In this study, berberine (BBR), a plant-derived isoquinoline alkaloid, exhibits multiple beneficial properties, including anti-inflammatory, antioxidant, and endothelial-protective effects, on the study group, perhaps by influencing VEGF concentration. Objective: This study aimed to investigate the effectiveness of BBR in addressing vascular function parameters linked to MAFLD, particularly its impact on serum VEGF levels and arterial stiffness. Methods: This randomized, double-blind, placebo-controlled clinical trial enrolled seventy individuals with MAFLD who were overweight or obese. Participants were randomly assigned in a 1:1 ratio to receive either BBR (1500 mg/day) or a placebo orally for 12 weeks. The following parameters were assessed pre- and post-intervention: VEGF, brachial SBP (Systolic Blood Pressure)/DBP (Diastolic Blood Pressure), MAP (Mean Arterial Pressure), AIx (Augmentation Index), AP (Aortic Pressure), number of waveforms, Pulse Pressure (PP), PWV (Pulse Wave Velocity), and PWA-SP/PWA-DP (Pulse Wave Analysis Systolic/Diastolic Pressure). The results for the metabolic parameters—FLI (Fatty Liver Index)—and anthropometric parameters—BMI (Body Mass Index), fat mass corp—and laboratory parameters, among them, hsCRP (high-sensitivity C-reactive protein), were published by us earlier. Results: In the BBR-treated cohort, VEGF concentrations demonstrated a statistically significant increase following the intervention, rising from a baseline mean of 456.23 ± 307.61 pg/mL to 561.22 ± 389.77 pg/mL (*p* < 0.0001). In the BBR group, a significant reduction in PWA-SP was observed after 12 weeks of supplementation (134.85 ± 16.26 vs. 124.46 ± 13.47 mmHg, *p* < 0.0001). No statistically significant differences were observed in the parameters determining arterial stiffness in the BBR and placebo groups. In the BBR group, delta VEGF correlated negatively with delta FLI; no such associations were observed in the placebo group. Changes in PWV were consistent and significantly correlated with changes in brachial SBP/DBP, PWA-SP, PWA-DP, and MAP. No serious adverse events were reported, and BBR was well tolerated. Conclusions: BBR appears to be a safe and promising adjunct in MAFLD therapy, potentially exerting reparative effects through VEGF modulation and vascular support. Further research is warranted to confirm its long-term impact and elucidate underlying protective mechanisms.

## 1. Introduction

The impact of BBR on anthropometric, hepatic, and metabolic parameters in patients with metabolic dysfunction–associated fatty liver disease has been discussed in our previous article [1]. In the present article, we aim to focus on the influence of BBR on vascular function parameters, among them VEGF, in individuals with MAFLD. Non-alcoholic fatty liver disease (NAFLD), and the proposed current term “metabolic dysfunction-associated fatty liver disease” (MAFLD), which better reflects the pathophysiology and cardiometabolic implications of this common liver disease, represents a significant global health burden. It is characterized by excessive hepatic fat accumulation, most often a consequence of abdominal obesity leading to insulin resistance, metabolic syndrome, and cardiovascular diseases. The consequences of progression of MAFLD may be Metabolic Dysfunction—Associated Steatohepatitis (MASH), fibrosis, cirrhosis, and hepatocellular cancer [2]. Understanding the molecular mechanisms underlying MAFLD is essential for developing effective therapeutic interventions.

Vascular endothelial growth factor (VEGF, also called VEGF-A) is a key regulator of angiogenesis, vascular permeability, and endothelial function. It belongs to the VEGF family and can activate cells via VEGF receptors, including VEGF-R1, VEGF-R2, VEGF-R3, and neurophilins. VEGF-R2 drives angiogenesis, leading to endothelial proliferation and new blood vessel formation, while VEGF-R1 negatively regulates this process. Dysregulated angiogenesis in pathological conditions can contribute to tumor growth and fibrosis [3]. While liver-derived molecules are known to impair vascular endothelial function, the extent of bidirectional communication between vascular endothelium and liver sinusoidal endothelial cells (LSECs) remains unclear. VEGF is a key regulator of hepatic microcirculation, and its dysregulation contributes to LSEC capillarization and fibrosis in MAFLD. LSEC damage induces hypoxia, activating hepatic stellate cells (HSCs) and promoting VEGF and angiopoietin signaling. Bone-marrow-derived endothelial progenitor cells (EPCs) also increase in chronic liver disease, potentially compensating for endothelial injury through VEGF-mediated crosstalk with LSECs. In MAFLD, VEGF-driven angiogenesis fuels chronic inflammation, while VEGF inhibition improves microcirculation and reduces liver inflammation in experimental models. Thus, targeting VEGF pathways may offer a therapeutic strategy to mitigate endothelial dysfunction and vascular risk in MAFLD. Overall, the endothelium could represent a “golden target” for the development of new treatment strategies for both conditions, with VEGF playing a pivotal role in disease progression and potential intervention [4]. However, VEGF’s role in the course of MAFLD remains complex, with both protective and pathological effects depending on disease stage and cellular environment [4].

BBR is a natural isoquinoline alkaloid derived from several medicinal plants, including Chinese goldthread (Coptis chinensis) and Barberry (Berberis vulgaris) [5]. BBR exerts profound effects on endothelial function by enhancing nitric oxide (NO) bioavailability, reducing oxidative stress, and modulating inflammatory pathways, thereby improving vascular homeostasis and mitigating endothelial dysfunction—a key factor in cardiovascular diseases. In the liver, BBR demonstrates hepatoprotective properties by regulating lipid and glucose metabolism, reducing hepatic steatosis, and exerting anti-inflammatory and antifibrotic effects. These mechanisms make BBR a promising therapeutic agent for conditions such as MAFLD [6]. VEGF modulation by BBR in humans with MAFLD remains unexplored. The impact of BBR on anthropometric and metabolic parameters in patients with MAFLD was comprehensively discussed in our previous publication [1]. Other authors have shown the atheroprotective effects of BBR by reducing endothelial dysfunction; inhibiting adhesion molecule expression, such as VCAM-1 (Vascular Cell Adhesion Molecule-1), ICAM-1 (Intercellular Adhesion Molecule-1), MCP-1 (Monocyte Chemoattractant Protein-1); preventing monocyte attachment; and suppressing vascular inflammation via AMPK activation. It also protects endothelial cells from apoptosis through modulation of the NF-κB (Nuclear Factor Kappa-Light-Chain-Enhancer of Activated B Cells), PI3K/Akt (phosphoinositide 3-kinase/Protein Kinase B), and MAPK (Mitogen-Activated Protein Kinase) pathways and enhances vascular function by upregulating eNOS expression and NO production. Furthermore, BBR mitigates oxidative stress by reducing reactive oxygen species (ROS) generation and strengthening antioxidant defenses [7,8]. The effect of BBR supplementation on VEGF concentration in obese patients with MAFLD has not yet been demonstrated.

VEGF signaling exhibits a dual, context-dependent role in MAFLD: while it can exert protective effects by promoting endothelial repair, angiogenesis, and microcirculatory homeostasis in early stages of the disease, persistent or excessive VEGF activation may contribute to pathological angiogenesis, hepatic stellate cell activation, and fibrosis progression. Highlighting this duality is essential for understanding the complex mechanisms of vascular–liver crosstalk and for evaluating interventions such as BBR that may modulate VEGF toward a protective rather than profibrotic outcome.

This study aims to investigate the effects of BBR supplementation on serum VEGF and other vascular function parameters in individuals with MAFLD. Using a randomized, double-blind, placebo-controlled trial design, we assess whether BBR influences VEGF concentration and other vascular parameters. The findings could provide insights into BBR’s therapeutic potential in modulating angiogenesis and vascular dysfunction in MAFLD patients, contributing to novel treatment strategies for this prevalent metabolic disorder.

## 2. Materials and Methods

### 2.1. The Study Design

This research was conducted as a randomized, double-blind, placebo-controlled clinical trial. Ethical approval was granted by the Bioethical Committee of Poznan University of Medical Sciences (no. 815/20). The study protocol was officially registered with the US National Institute of Health under ClinicalTrials.gov (Identifier: NCT05523024). Participant recruitment took place at the outpatient department of the University Hospital in Poznan, Poland. Prior to enrollment, all participants provided written informed consent to participate in the study.

#### 2.1.1. Participants

After the screening process, 70 patients with MAFLD were invited to join the study.

#### 2.1.2. Inclusion Criteria

Participants met the following conditions:

- Diagnosis of MAFLD (as per the 2020 criteria [9]), which requires hepatic steatosis plus at least one of the following: overweight/obesity or metabolic dysregulation in lean individuals. Hepatic steatosis was confirmed via ultrasound (patients were enrolled based on a prior diagnosis of hepatic steatosis, confirmed by ultrasound examination).
- Body Mass Index (BMI) between 27.0 kg/m^2^ and 34.9 kg/m^2^
- Abdominal obesity, defined by waist circumference >80 cm for women and >94 cm for men according to the International Diabetes Federation [10].
- Age between 40 and 60 years.
- Women who were at least one year post-menopause.
- Stable body weight for the three months prior to the study (within ±3 kg).

#### 2.1.3. Exclusion Criteria

Participants were excluded if they had any of the following:

- A history of following alternative diets in the three months before the study.
- Use of dietary supplements within three months before the study.
- Recent intake (past three months) of antibiotics, probiotics, or prebiotics.
- Secondary obesity, prior bariatric surgery, or pharmacological treatment for obesity within three months of the study.
- Other liver diseases, including high risk of NASH (Fibrosis-4 (FIB-4) Index for Liver Fibrosis > 2.67), autoimmune hepatitis, hepatitis B or C, toxic hepatitis, cirrhosis, Wilson’s disease, or hemochromatosis.
- Gastrointestinal disorders such as inflammatory bowel disease (IBD), celiac disease, gastritis, duodenitis, pancreatic disorders, or symptoms indicative of irritable bowel syndrome (IBS).
- Acute inflammatory conditions with elevated high-sensitivity C-reactive protein (hsCRP).
- Impaired kidney function—Glomerular Filtration Rate (GFR) (<60 mL/min/1.73 m^2^).
- Type 2 diabetes mellitus (T2DM).
- Dyslipidemia or hypertension requiring the initiation or modification of pharmacological treatment within six months before the study or during the intervention. First-degree hypertension managed with a single medication and dyslipidemia treated with monotherapy (excluding statins) were acceptable.
- Chronic pharmacotherapy, including nonsteroidal anti-inflammatory drugs (NSAIDs), proton pump inhibitors, anticoagulants, or medications known to affect metabolism (e.g., second-generation antipsychotics).
- Conditions requiring specific dietary management or long-term supplementation.
- Alcohol consumption exceeding 30 g/day for men or 20 g/day for women, nicotine dependence, or substance abuse.
- Mental health disorders, including eating disorders.
- Cancer or autoimmune diseases.
- Use of hormone replacement therapy (HRT) at the time of study enrollment or during the study period.
- Any condition that could interfere with study outcomes or pose a risk to participants’ health.

#### 2.1.4. Participant Allocation and Berberine Supplement

A total of 70 eligible patients were enrolled in the study after meeting the inclusion criteria and having no exclusion factors. Participants were randomly assigned to one of two groups, with the randomization process overseen by an independent statistician to ensure unbiased allocation. Both the investigators and participants were blinded to group assignments. Each participant was assigned a unique identifier code and was allocated in a 1:1 ratio to either the placebo or the BBR group. The subjects’ randomization codes were concealed until the statistical analysis. Blinding was ensured by using identical, opaque, unlabeled, tightly sealed, white capsules for both BBR and placebo so that neither the investigators nor the participants could distinguish between the treatments.

**Figure 1:**
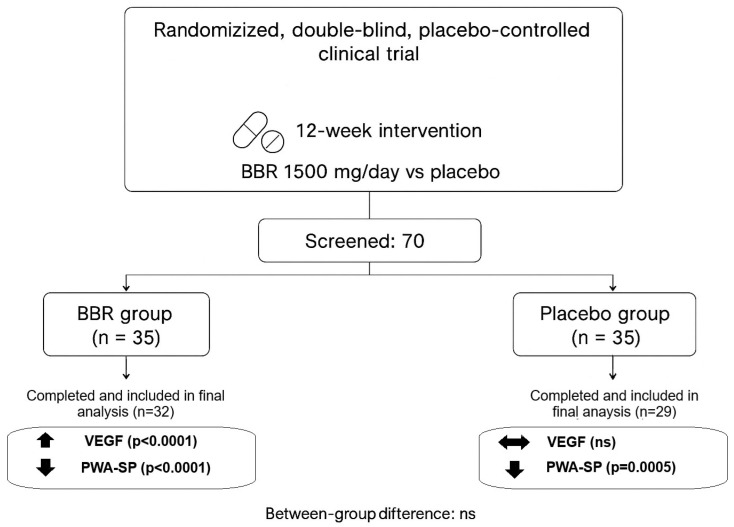
Combined flowchart of study design, participant flow, and key outcomes (VEGF, PWA-SP).

Figure 1 presents the study flowchart.

Group 1 (*n* = 35) received berberine (berberine hydrochloride 97% extract from Berberis aristata), administered at a total daily dose of 1500 mg, divided into three doses.

Group 2 (*n* = 35) received a placebo, administered in three doses.

The BBR supplement was taken orally for 12 weeks, with participants consuming two capsules three times a day before meals:

Morning dose: 7:00–9:00 a.m. (before breakfast);

Afternoon dose: 1:00–2:00 p.m. (before lunch);

Evening dose: 6:00–7:00 p.m. (before dinner).

The placebo followed the same administration method and dosage. It contained only excipients (potato starch) and was administered in the same manner over a 12-week period, with both groups consuming six capsules daily—two capsules three times per day at fixed times.

#### 2.1.5. Adverse Effect, Safety, and Compliance

It is important to highlight that in some cases, BBR supplementation led to mild and temporary gastrointestinal symptoms during the initial days or weeks of treatment. Mild gastrointestinal symptoms, such as bloating or a bitter aftertaste, occurred but did not require adjustments to the supplementation regimen. Throughout the 12-week intervention, no serious adverse events (SAEs) were observed. The most frequently reported side effects included flatulence and a bitter aftertaste, but these symptoms did not necessitate medical intervention or result in any health deterioration. The tolerability of berberine at a daily dose of 1500 mg was deemed acceptable.

Regarding study adherence, three participants from the BBR group were excluded from the final analysis due to insufficient compliance with the study protocol. In the placebo group, five individuals did not complete the study (due to personal reasons), and one participant was excluded due to non-compliance with study requirements (Figure 1).

#### 2.1.6. Safety Profile

The data on safety are presented in Table 1.

#### 2.1.7. Medical Data

At the initial visit, participants underwent an assessment of their socio-demographic and medical background, which included completing a questionnaire, a medical history interview, and a review of medical records. To ensure compliance with the study regimen, participants were given a medication journal to track their intake, record any missed doses, and note any potential side effects. To monitor study adherence and detect any potential adverse effects, remote consultations were conducted at the study’s midpoint. During the initial visit, each participant received a 12-week supply of the assigned supplement. On the final visit, they returned their empty packaging along with a medication journal to monitor adherence.

#### 2.1.8. Diet and Physical Activity Evaluation

As part of the Diet and Physical Activity Evaluation, standardized questionnaires were employed to monitor and ensure consistency of lifestyle conditions throughout the intervention period.

At both the baseline (enrollment and group allocation) and the study endpoint, assessments were conducted to evaluate anthropometric measurements, body composition, dietary intake, and physical activity levels.

To minimize the risk of bias due to changes in diet or physical activity, participants were advised to maintain their usual dietary habits and physical activity levels throughout the study. Compliance with these instructions was monitored through dietary and physical activity assessments, which were conducted before the intervention, during remote follow-up visits, and at the study’s conclusion. We used standardized tools: the Food Frequency Questionnaire (FFQ-6) and the International Physical Activity Questionnaire (IPAQ).

#### 2.1.9. Anthropometric Measurements

Anthropometric Measurements were essential criteria for participant qualification in the study.

Body weight and height—Weight was measured with an accuracy of 0.1 kg, while height was recorded to the nearest 0.5 cm.

Body Mass Index (BMI)—Calculated as weight (kg) divided by height squared (m^2^).

Obesity is characterized by excessive fat accumulation, surpassing levels considered optimal for health. BMI is a widely used index for evaluating body weight relative to height. Another method for identifying obesity and quantifying body fat percentage is BIA, used in an earlier publication.

#### 2.1.10. Calculated Parameters—Formulae

FLI = (e0.953 × loge (TG) + 0.139 × BMI + 0.718 × loge (GGT) + 0.053 × WC − 15.745)/(1 + e0.953 × loge (TG) + 0.139 × BMI + 0.718 × loge (GGT) + 0.053 × WC − 15.745) × 100(1)

#### 2.1.11. Functional Parameters

Key physiological measurements were taken at both baseline and the study endpoint, including Systolic Blood Pressure (SBP) and Diastolic Blood Pressure (DBP). Additionally, Pulse Wave Velocity (PWV) and Pulse Wave Analysis (PWA) were performed using the SphygmoCor system (Atcor Medical Blood Pressure Analysis System, Sydney, Australia), assessing parameters such as brachial SBP, brachial DBP, systolic and diastolic pressure (PWA-SP, PWA-DP), number of waveforms, Mean Arterial Pressure (MAP), Pulse Pressure (PP), Augmentation Index (AIx), and Aortic Pressure (AP). PWV and PWA were conducted using the SphygmoCor system in a temperature-controlled setting before performing anthropometric measurements and collecting blood samples. PWV was measured between the carotid and femoral arteries, with participants lying in a supine position. Pulse wave recordings were obtained non-invasively using a SphygmoCor probe placed over both the carotid and femoral arteries, while an electrocardiogram (ECG) was simultaneously recorded. To ensure an artifact-free ECG signal, the skin was prepared properly by removing hair at the electrode placement sites and cleansing the area with an alcohol wipe. Once a stable, accurate, and reproducible pulse wave signal was detected, at least 12 s of ECG data (approximately 10 heartbeats) was recorded. The distance between the carotid and femoral arteries was measured directly, using the suprasternal notch as a reference point, and the values were entered into the AtCor Medical SphygmoCor-XCEL (www.cardiex.com; accessed on 5 October 2025). PWV was determined by calculating the time delay between specific timing points on two pressure waveforms measured at a known distance apart. The SphygmoCor method identifies the foot of the waveform as the onset marker to determine time differences between the R-wave of the ECG and the pulse waveforms at each measurement site. The AtCor Medical SphygmoCor-XCEL software then automatically calculated PWV as the carotid–femoral artery distance divided by the pulse wave travel time. Only PWV values with a standard deviation below 10% were included in the final analysis. Additionally, central aortic hemodynamic parameters, including AIx, AP, and PP, were assessed using applanation tonometry of the radial artery, following standard procedures. The systolic phase of the central arterial waveform is characterized by two distinct pressure peaks: the first peak results from blood ejected by the left ventricle. The second peak is caused by wave reflections from peripheral arteries. The AP represents the absolute increase in PP due to wave reflection [11].

These assessments of functional parameters took place at the Department of Treatment of Obesity, Metabolic Disorders and Clinical Dietetics, Poznań University of Medical Sciences, Poland.

#### 2.1.12. Laboratory Tests

Blood samples were collected following an overnight fasting and rest period, adhering to the standards set by the International Organization for Standardization (ISO). The serum level of VEGF was measured using a quantitative immunoassay (R&D Systems, Minneapolis, MN, USA).

### 2.2. Statistical Analysis

All statistical computations and analyses were conducted using Statistica, version 13 (TIBCO Software Inc., 2017, Palo Alto, CA, USA). Quantitative variables were expressed as mean ± standard deviation, as well as median, minimum, and maximum values. The Shapiro–Wilk test was applied to assess the normality of data distribution. In cases where variables followed a normal distribution, Student’s *t*-test for independent samples was used to compare the placebo group with the group receiving berberine, while the paired Student’s *t*-test was used to evaluate the treatment effect within each group. Since most variables did not follow a normal distribution, non-parametric tests were primarily utilized. The Mann–Whitney U test was applied for comparisons between independent groups, and the Wilcoxon signed-rank test was used for comparisons within groups before and after the intervention. Correlations between selected variables were assessed using Spearman’s rank correlation coefficient or the Pearson correlation test. A *p*-value of less than 0.05 was considered statistically significant for all tests.

Post-intervention vascular parameters (PWV, AIx, and PWA-SP) were additionally analyzed using analysis of covariance (ANCOVA), with age and the respective baseline values entered as covariates to control for potential confounding. Adjusted means and corresponding *p*-values were reported for between-group comparisons.

The sample size calculation was based on data from previous interventional studies evaluating VEGF changes in metabolic disorders and MAFLD. In particular, mean baseline and post-treatment VEGF concentrations and standard deviations reported by previous reports were used as reference values. Assuming a moderate expected change (corresponding approximately to Cohen’s d ≈ 0.6), a significance level of α = 0.05, and a power of 0.8, the minimum sample size was estimated as 28 subjects per group. To compensate for expected dropouts, 35 participants were enrolled in each group.

## 3. Results

The baseline characteristics of the BBR and placebo groups are presented in Table 2. There were no significant differences between the groups, except for age. However, in both groups, age was within the range specified by the enrollment criteria. After adjustment for baseline values and age using ANCOVA, a significant group effect was detected for PWV (*p* < 0.0001) and PWA-SP (*p* < 0.0001), whereas the difference in AIx did not reach statistical significance (*p* = 0.0707). This indicates that group differences in PWV and PWA-SP became evident only after controlling for age and baseline variability.

Post-intervention characteristics did not differ significantly between the study groups (Table 3).

Our results demonstrate a statistically significant increase in VEGF levels following BBR supplementation, compared to the baseline. VEGF concentration in the BBR group before intervention was 456.23 ± 307.61 pg/mL, and after 12 weeks, it was 561.22 ± 389.77 pg/mL, *p*-value < 0.0001. In the BBR group, a statistically significant decrease in PWA-SP levels was observed between baseline and after 12 weeks of BBR supplementation: PWA-SP measured in mmHg was 134.85 ± 16.26 before and 124.46 ± 13.47 after, *p*-value < 0.0001. Additionally, a decrease in PWA-SP was noted within the placebo group after the intervention (*p* = 0.0005). The data are presented in Table 4.

The study parameters did not show statistically significant differences in changes after the 12-week intervention. Changes in vascular and hemodynamic parameters in the BBR and placebo groups are presented in Table 5.

Effect sizes (Cohen’s d) were calculated post hoc to complement the interpretation of the observed changes. As shown in Table 6, the within-group effect for VEGF in the BBR group was large (*d* = 0.94), while all vascular parameters, including PWA-SP, showed negligible between-group effects.

In the BBR group, the direction of change in delta VEGF was negatively correlated with the direction of change in delta FLI (*r* = −0.42) (*p* < 0.05), whereas no such association was observed in the placebo group.

Furthermore, in the intervention group, changes in PWV were consistent and significantly correlated with changes in brachial SBP (*r* = 0.36), brachial DBP (*r* = 0.51), PWA-SP (*r* = 0.42), PWA-DP (*r* = 0.50), and MAP (*r* = 0.51). The data are presented in Table 7.

## 4. Discussion

The study provided novel insights into the role of VEGF in MAFLD, particularly in the context of BBR supplementation, highlighting its potential involvement in vascular modulation and hepatic repair mechanisms.

VEGF is a signal protein that plays a crucial role in angiogenesis, vasculogenesis, and endothelial cell function. It is primarily responsible for promoting the formation of new blood vessels (neovascularization) and increasing vascular permeability. Angiogenesis is often triggered by hypoxia and inflammation, which are common in liver diseases, including MAFLD [12,13]. However, the overall impact of VEGF in MAFLD is context-dependent, as excessive angiogenesis can contribute to disease progression and complications like fibrosis, while, on the other hand, an increase in VEGF may potentially improve hepatocyte function in the initial phase of steatosis [13].

Our study supports the hypothesis that VEGF may play a protective role in MAFLD, particularly in its early stages. We observed that BBR administration significantly increased VEGF levels, suggesting that BBR may exert a reparative effect through the modulation of VEGF concentration.

Generally, VEGF is elevated in MAFLD, with higher levels in NASH. VEGF also influences lipogenesis and fibrogenesis, affecting liver disease progression. Targeting VEGF or its receptors could impact both angiogenesis and fibrosis, offering potential therapeutic strategies. However, since angiogenesis may aid fibrosis resolution, VEGF inhibition must be carefully considered in treatment approaches [13]. Interestingly, one study found no elevation in VEGF levels in the serum of MAFLD patients, in contrast to vascular cell adhesion molecule-1 (VCAM-1), which did show increased levels and is indicative of endothelial activation [14]. Recent experimental studies suggest that VEGF plays a reparative role in liver regeneration by promoting the recruitment and differentiation of biliary epithelial progenitor cells (BECs) into hepatocytes. This mechanism becomes especially relevant when hepatocyte proliferation is impaired due to chronic injury. In animal models, VEGF overexpression enhances BEC-to-hepatocyte conversion and improves liver pathology, including reductions in steatosis and fibrosis. These findings highlight VEGF as a potential therapeutic target for supporting liver repair in MAFLD [15]. In previous studies, serum VEGF levels tended to be lower in patients with MAFLD compared to healthy controls and were significantly reduced in those with NASH. This suggests that a decline in VEGF may be associated with disease progression. Therefore, increasing VEGF levels in MAFLD/MASH—toward those seen in healthy individuals—might reflect a shift toward a more reparative, less fibrotic liver environment [16]. In the present study, a negative correlation was observed between changes in VEGF and changes in FLI, meaning that as VEGF increased, FLI decreased. This may indicate a compensatory mechanism whereby VEGF rises in response to less pronounced FLI improvement, potentially enhancing regenerative processes and acting synergistically with BBR. However, this hypothesis requires confirmation in studies of longer duration and with larger sample sizes.

In our study, a correlation involving hsCRP was detected only in the placebo group. Here, an inverse relationship between delta VEGF and delta CRP was noted, suggesting that a greater increase in VEGF was associated with a smaller change in CRP. This could imply that an increase in VEGF is not associated with a heightened inflammatory response in the early stage of MAFLD. However, such a relationship was not observed in the BBR group, which limits this interpretation. The correlation between VEGF and CRP in the placebo group may suggest that VEGF acts via an anti-inflammatory mechanism, counteracting transient inflammatory states independently of supplementation.

BBR plays a key role in controlling vascular smooth muscle cells by stopping their overgrowth and movement, which helps prevent plaque buildup. With these combined effects, BBR acts as a powerful protector of cardiovascular health [17]. BBR also suppresses foam cell formation through AMPK (Activated Protein Kinase)/SIRT1 (Sirtuin 1)/PPARγ (peroxisome proliferator-activated receptor gamma) activation. Computational studies confirm BBR’s strong binding to cardiovascular disease targets, highlighting its therapeutic potential [18,19].

Our study findings align with several known mechanisms while providing novel insights. BBR supplementation resulted in a reduction in PWA-SP similar to the placebo group. BBR supplementation appears to have a beneficial effect on hemodynamic parameters but does not significantly change PWV. This lack of effect on arterial stiffness may be attributable to the relatively short duration of the intervention—12 weeks may be insufficient to induce measurable improvements in vascular elasticity with BBR. Changes in delta PWV in the BBR group corresponded with shifts in vascular parameters (brachial SBP, brachial DBP, PWA-SP, PWA-DP, and MAP), indicating that greater reductions in PWV were associated with parallel improvements in vascular function, likely driven by BBR supplementation, as we did not observe such changes in the placebo group. The ANCOVA revealed a significant between-group difference in PWV and PWA-SP after adjusting for baseline values and age, suggesting that age-related variability may have masked this effect in the unadjusted analysis. Therefore, the observed difference should be interpreted as age-adjusted rather than a purely treatment-specific effect. The observed correlations between PWV and various blood pressure parameters, as well as other hemodynamic indices, are likely physiological in nature, reflecting the inherent relationship between metabolic changes and vascular stiffness. It is possible that a longer course of BBR therapy could correct these parameters; however, this effect was not evident after 12 weeks. BBR effectively improves vascular health in hypertensive rats by preserving endothelial integrity and reducing arterial stiffness. BBR treatment lowered circulating endothelial microparticles and enhanced endothelial progenitor cell numbers and function, which correlated with improved endothelium-dependent vasodilation and reduced aortic PWV. These findings suggest that BBR may protect against vascular injury in hypertension, potentially by modulating endothelial repair mechanisms and factors, such as VEGF, that are critical for maintaining endothelial function and arterial elasticity [20]. In fact, BBR also induced mobilization of endothelial progenitors and positively influenced vascular elasticity in human patients [21]. Regarding the liver, a study conducted on a thioacetamide-induced mouse model of liver fibrosis demonstrated that a hematopoietic stem cell mobilizer—Stem Enhance, derived from cyanophyta Aphanizomenon flos-aquae—also positively influenced the number of CD34+ cells and decreased hepatic fibrosis, which accompanied the upregulation of VEGF expression [22]. Both BBR and Stem Enhance act through similar biochemical pathways (AMPK, NF-κB) and share anti-inflammation and cardiovascular protective properties [23]. Thus, the tentative hypothesis may link the mechanism of regenerative potential of VEGF with the recruitment of hematopoietic progenitor cells from the circulation; however, further studies are required to confirm this.

Due to the application of very strict inclusion and exclusion criteria, we obtained a homogeneous, highly selected group, which unfortunately limited its size. As commonly observed in intervention studies, the number of participants decreased further during the trial, affecting the final comparison of outcomes. Additionally, the 12-week duration of the BBR intervention may have been insufficient to detect changes in certain vascular parameters, such as PWV. The obtained results can be considered a pilot, providing a basis for a larger, more comprehensive trial in the future.

Our study limitations include the poor bioavailability of BBR, which remains an unresolved issue. Although patient compliance was high, adhering to a three-times daily regimen may be difficult for some. Future studies should consider longer intervention periods and larger sample sizes to better assess outcomes. Additionally, investigating the impact of higher BBR doses and their relationship with time and effectiveness in patients with MAFLD is essential. The innovative aspect of this study lies in the assessment of dynamic endothelial function parameters (PWA and PWV), alongside VEGF levels in individuals with MAFLD following BBR supplementation. To date, no studies have combined these vascular biomarkers with angiogenic profiling in the context of MAFLD and BBR intervention, making our findings unique and novel in the current scientific landscape.

## 5. Conclusions

This randomized, double-blind, placebo-controlled trial suggests that BBR supplementation beneficially modulates VEGF levels and improves vascular parameters in MAFLD patients. Given the dual role of VEGF—protective in early NAFLD and pathogenic in advanced disease—these findings highlight the need for further research into BBR’s regulatory effects on endothelial function, inflammation, and fibrosis. BBR was well tolerated and demonstrated pleiotropic benefits, supporting its potential as an adjunctive therapy for MAFLD, particularly in cardiovascular risk reduction. Screening for endothelial dysfunction in MAFLD could help identify patients at higher risk for adverse hepatic and cardiovascular outcomes. Larger studies are necessary to validate these findings and clarify the underlying mechanisms.

From a clinical perspective, these results may contribute to more personalized management of MAFLD by integrating endothelial markers into risk stratification and by supporting the therapeutic use of BBR in patients with early-stage disease and coexisting cardiometabolic disturbances. This approach can assist in the individualization of metabolic treatment strategies.

## Figures and Tables

**Table 1:** Safety profile and composition of berberine supplement and placebo.

Parameter	Berberine Supplement (BBR)	Placebo
Safety compliance	Compliant with all regulatory safety standards	Compliant with all regulatory safety standards
Heavy metal content		
Lead	≤3 ppm (µg/g)	≤3 ppm (µg/g)
Arsenic	≤1 ppm (µg/g)	≤1 ppm (µg/g)
Cadmium	≤1 ppm (µg/g)	≤1 ppm (µg/g)
Mercury	≤0.1 ppm (µg/g)	≤0.1 ppm (µg/g)
Residual pesticides	Not applicable	Not applicable
Polycyclic Aromatic hydrocarbons		
Benzo(a)pyrene	≤10 ppb (µg/kg)	≤10 ppb (µg/kg)
PAH4 (sum of 4 PAHs)	≤50 ppb (µg/kg)	≤50 ppb (µg/kg)
Microbiological testing		
Total aerobic microbial count	≤5000 cfu/g	≤5000 cfu/g
Yeasts and molds	≤100 cfu/g	≤100 cfu/g
*Escherichia coli*	Negative/10 g	Negative/10 g
*Salmonella*	Negative/25 g	Negative/25 g
*Staphylococcus aureus*	Negative/10 g	Negative/10 g
*Pseudomonas aeruginosa*	Negative/10 g	Negative/10 g
Bile-tolerant Gram-negative bacteria	Negative/10 g	Negative/10 g
Coliforms	<10 cfu/g	<10 cfu/g
Additional Information		
GMO status	Free from genetically modified organisms	Free from genetically modified organisms
BSE/TSE status	Free from BSE/TSE	Free from BSE/TSE
Dietary compatibility	Gluten-free, lactose-free, suitable for vegans	Gluten-free, lactose-free, suitable for vegans
Manufacturing and supply		
Manufacturer	Sami Labs Ltd., Bangalore, India	Standard Sp. z o.o., Lublin, Poland
Supplier	Sabinsa Poland Sp. z o.o., Lubon, Poland	Not applicable
Substance name	Berberine extract	Potato starch
Chemical formula	C_20_H_18_NO_4_	(C_6_H_10_O_5_)_n_
CAS number	2086-83-1	9005-84-9
Molar mass	336.36 g/mol	Not specified (polymer)

**Table 2:** Baseline characteristics.

Variable	BBR (*n* = 32)	Mean ± SD	Median	Min–Max	PLACEBO (*n* = 29)	Mean ± SD	Median	Min–Max	*p* (Between Groups)
Age		49.09 ± 5.00	50.0	40.00–59.00		53 ± 6.25	52.5	42.00–60.00	**0.0175**
BMI [kg/m^2^]		32.22 ± 3.46	31.7	27.80–35.90		30.90 ± 3.55	29.8	27.80–34.80	0.0975
VEGF [pg/mL]		456.23 ± 307.61	402.26	110.45–1739.65		414.51 ± 230.48	359.32	25.25–882.96	0.8569
PWA-SP [mmHg]		134.85 ± 16.26	133.00	110.00–175.00		132.29 ± 12.65	133.00	105.00–154.00	0.4870 *
PWA-DP [mmHg]		83.61 ± 9.17	83.00	68.00–100.00		80.81 ± 11.14	81.00	56.00–106.00	0.5970 *
Brachial SBP [mmHg]		135.63 ± 16.11	134.00	110.00–175.00		133.32 ± 13.80	133.50	105.00–165.00	0.7213
Brachial DBP [mmHg]		84.00 ± 9.04	84.00	68.00–100.00		81.89 ± 10.80	82.50	56.00–106.00	0.3944
MAP		100.74 ± 10.34	101.00	84.00–118.00		99.74 ± 11.31	99.50	77.00–125.00	0.6887
PP		40.11 ± 9.68	39.00	28.00–63.00		39.97 ± 9.28	39.00	17.00–63.00	0.7880
AP		13.34 ± 7.37	12.00	0.00–34.00		15.03 ± 10.46	12.50	−2.00–52.00	0.6487
AIx		31.57 ± 13.72	29.00	0.00–66.00		35.26 ± 22.70	35.00	−10.00–131.00	0.3883
PWV [m/s]		7.31 ± 1.31	7.00	5.00–10.40		6.89 ± 2.01	6.90	2.30–12.30	0.2635
hsCRP		0.32 ± 0.24	0.27	0.04–1.02		0.30 ± 0.30	0.22	0.03–1.58	0.3952
FLI		68.49 ± 25.58	75.90	16.20–99.30		58.18 ± 25.24	55.60	16.00–97.90	0.0948

Abbreviations: BMI—Body Mass Index; VEGF—Vascular Endothelial Growth Factor; PWV—Pulse Wave Velocity; PWA-SP—Pulse Wave Analysis Systolic Pressure; PWA-DP—Pulse Wave Analysis Diastolic Pressure; brachial SBP—Brachial Systolic Blood Pressure; brachial DBP—Brachial Diastolic Blood Pressure; MAP—Mean Arterial Pressure, PP—Pulse Pressure; AP—Aortic Pressure; AIx—Augmentation Index; FLI—Fatty Liver Index; hsCRP—high-sensitivity C-reactive protein; standard deviation; *p*—*p*-value (statistical significance). Bold text indicates statistical significance: *p*-value < 0.05. * Parametric Statistical Tests.

**Table 3:** Post-intervention characteristics.

Parameter	BBR (*n* = 32)	Mean ± SD	Median	Min–Max	Placebo (*n* = 29)	Mean ± SD	Median	Min–Max	*p* (Between Groups)
VEGF [pg/mL]		561.22 ± 389.77	522.84	149.12–2037.87		421.79 ± 270.15	362.11	102.19–1352.21	0.0638
PWA-SP [mmHg]		124.46 ± 13.47	124.00	98.00–154.00		122.66 ± 14.82	119.00	101.00–167.00	0.8102
PWA-DP [mmHg]		85.20 ± 10.41	83.00	58.00–115.00		83.23 ± 10.38	80.00	64.00–116.00	0.1976
Brachial SBP [mmHg]		134.49 ± 14.79	133.00	107.00–168.00		132.26 ± 17.32	128.00	107.00–185.00	0.3618
Brachial DBP [mmHg]		84.31 ± 10.58	82.00	56.00–115.00		82.06 ± 10.11	80.00	64.00–116.00	0.1493
MAP		100.54 ± 11.50	100.00	74.00–132.00		98.69 ± 11.05	96.00	85.00–133.00	0.2714
PP		39.34 ± 8.90	39.00	21.00–65.00		39.69 ± 10.73	38.00	25.00–72.00	0.7974
Ap		13.29 ± 6.68	12.00	3.00–32.00		14.88 ± 12.02	11.00	−2.00–65.00	0.9354
AIx		32.54 ± 11.62	33.00	10.00–56.00		35.56 ± 23.47	33.00	−7.00–129.00	0.6400
PWV [m/s]		7.61 ± 1.09	7.50	6.10–11.20		6.77 ± 2.03	6.60	6.70–10.10	0.7801
hsCRP		0.39 ± 0.42	0.28	0.03–2.00		0.26 ± 0.21	0.15	0.03–0.71	0.2633
FLI		67.4 ± 25.68	78.10	14.20–98.60		61.70 ± 25.61	67.10	17.21–100.00	0.3681

Abbreviations: VEGF—Vascular Endothelial Growth Factor; PWA-SP—Pulse Wave Analysis Systolic Pressure; PWA-DP—Pulse Wave Analysis Diastolic Pressure; brachial SBP—Brachial Systolic Blood Pressure; brachial DBP– Brachial Diastolic Blood Pressure; PWV—Pulse Wave Velocity; MAP—Mean Arterial Pressure; PP—Pulse Pressure; AP—Aortic Pressure, AIx—Augmentation Index; FLI—Fatty Liver Index; hsCRP—high sensitivity C-reactive protein; SD—standard deviation; *p*—*p*-value (statistical significance).

**Table 4:** Baseline and post-intervention (after 12 weeks) outcomes.

		BBR				Placebo
	Mean ± SD		*p*-Value		Mean ± SD	*p*-Value
	Before	After		Before	After	
VEGF [pg/mL]	456.23 ± 307.61	561.22 ± 389.77	**<0.0001**	414.51 ± 230.48	421.79 ± 270.15	0.3591
PWA-SP [mmHg]	134.85 ± 16.26	124.46 ± 13.47	<0.0001 *	132.29 ± 12.65	122.66 ± 14.82	**0.0005**
PWA-DP [mmHg]	83.61 ± 9.17	85.2 ± 10.41	0.5866 *	80.81 ± 11.14	83.23 ± 10.38	0.2301
Brachial SBP [mmHg]	135.63 ± 16.11	134.49 ± 14.79	0.4167	133.32 ± 13.80	132.26 ± 17.32	0.5071
Brachial DBP [mmHg]	84.00 ± 9.04	84.31 ± 10.58	0.9869	81.89 ± 10.80	82.06 ± 10.11	1
MAP	100.74 ± 10.34	100.54 ± 11.50	0.9288 *	99.74 ± 11.31	98.69 ± 11.05	0.4082
PP	40.11 ± 9.68	39.34 ± 8.90	0.4022	39.97 ± 9.28	39.69 ± 10.73	0.9935
AP	13.34 ± 7.37	13.29 ± 6.68	0.7791	15.03 ± 10.46	14.88 ± 12.02	0.8457
AIx	31.57 ± 13.72	32.54 ± 11.62	0.7477	35.26 ± 22.70	35.56 ± 23.47	0.7223
PWV [m/s]	7.31 ± 1.31	7.61 ± 1.09	0.0984	6.89 ± 2.01	6.77 ± 2.03	0.8122
hsCRP	0.32 ± 0.24	0.39 ± 0.42	0.4237	0.30 ± 0.30	0.26 ± 0.21	0.4549
FLI	68.49 ± 25.58	67.4 ± 25.68	0.9766	58.18 ± 25.24	61.70 ± 25.61	0.6766

Abbreviations: VEGF—Vascular Endothelial Growth Factor; brachial SBP—Brachial Systolic Blood Pressure [mmHg]; brachial DBP—Brachial Diastolic Blood Pressure [mmHg]; MAP—Mean Arterial Pressure [mmHg]; AIx—pulse Wave Analysis Augmentation Index; PP—Pulse Pressure; AP—Aortic Pressure; PWA-DP—Pulse Wave Analysis Diastolic Pressure [mmHg]; PWA-SP—Pulse Wave Analysis Systolic Pressure [mmHg]; PWV—Pulse Wave Velocity [m/s]; FLI—Fatty Liver Index; hsCRP—high-sensitivity C-reactive protein; SD—standard deviation; *p*—*p*-value (statistical significance). Bold text indicates statistical significance: *p*-value < 0.05. * Parametric Statistical Tests.

**Table 5:** Changes in vascular and hemodynamic parameters in the berberine and placebo groups.

Parameter	BBR (*n* = 32)	Berberine Mean ± SD	Berberine Median (Min–Max)	Placebo (*n* = 29)	Placebo Mean ± SD	Placebo Median (Min–Max)	*p*-Value (Between Groups)
ΔVEGF [pg/mL]		104.99 ± 111.76	73.64 (−89.28–454.66)		76.68 ± 125.17	57.27 (−62.68–548.02)	0.1090
ΔBrachial SBP [mmHg]		−1.14 ± 13.52	−1.00 (−27.00–44.00)		−1.51 ± 13.44	−1.00 (−33.00–24.00)	0.9085 *
ΔBrachial DBP [mmHg]		0.31 ± 9.90	−2.00 (−19.00–27.00)		0.09 ± 7.95	−1.00 (−15.00–18.00)	0.9155 *
ΔPWV [m/s]		0.29 ± 0.88	0.25 (−1.30–2.00)		−0.11 ± 2.03	−0.70 (−6.40–3.00)	0.6121 *
ΔPWA-SP [mmHg]		−0.40 ± 11.62	−1.00 (−23.00–38.00)		−1.06 ± 10.13	−1.00 (−28.00–19.00)	0.8017 *
ΔPWA-DP [mmHg]		0.43 ± 9.64	−1.00 (−18.00–24.00)		−0.14 ± 8.47	0.00 (−16.00–19.00)	0.7930
ΔMAP		0.29 ± 9.52	−1.50 (−20.00–20.00)		−1.29 ± 9.43	−1.00 (−16.00–16.00)	0.4910 *
ΔPP		−0.77 ± 11.05	−2.00 (−22.00–37.00)		−0.60 ± 9.03	−1.00 (−34.00–14.00)	0.5912
ΔAP		−0.06 ± 6.85	0.00 (−13.00–22.00)		−0.34 ± 13.76	0.50 (−43.00–51.00)	0.7600
ΔAIx		0.97 ± 11.59	0.00 (−19.00–30.00)		0.43 ± 14.79	2.00 (−32.00–36.00)	0.9012
ΔhsCRP		−0.07 ± 0.46	0.02 (−1.85–0.68)		−0.02 ± 0.13	0.00 (−0.41–0.32)	0.2560
ΔFLI		−0.46 ± 12.54	−0.10 (−31.9–26.00)		−1.76 ± 11.11	0.25 (−30.90–18.77)	0.9479

Abbreviations: VEGF—Vascular Endothelial Growth Factor [pg/mL]; PWV—Pulse Wave Velocity [m/s]; PWA-SP—Pulse Wave Analysis Systolic Pressure [mmHg]; PWA-DP—Pulse Wave Analysis Diastolic Pressure [mmHg]; brachial SBP—Brachial Systolic Blood Pressure [mmHg]; brachial DBP—Brachial Diastolic Blood Pressure [mmHg]; MAP—Mean Arterial Pressure; PP—Pulse Pressure; AP—Aortic Pressure; AIx—Augmentation Index; FLI—Fatty Liver Index; hsCRP—high-sensitivity C-reactive protein; SD—standard deviation; *p*—*p*-value (statistical significance). * Parametric Statistical Tests.

**Table 6:** Effect sizes (Cohen’s d) for within- and between-group changes.

Parameter	d (Within BBR)	d (Within Placebo)	d (Between Groups)
ΔVEGF [pg/mL]	0.94	0.61	0.24
ΔBrachial SBP [mmHg]	−0.08	−0.11	0.03
ΔBrachial DBP [mmHg]	0.03	0.01	0.02
ΔPWV [m/s]	0.33	−0.05	0.26
ΔPWA-SP [mmHg]	−0.03	−0.10	0.06
ΔPWA-DP [mmHg]	0.05	−0.02	0.06
ΔMAP [mmHg]	0.03	−0.14	0.17
ΔPP [mmHg]	−0.07	−0.07	−0.02
ΔAP [mmHg]	−0.01	−0.03	0.03
ΔAIx	0.08	0.03	0.04
ΔhsCRP	−0.15	−0.15	−0.15
ΔFLI	−0.04	−0.16	0.11

d—Cohen’s d represents the standardized mean difference, calculated as the mean change divided by the pooled standard deviation of change scores.

**Table 7:** Changes (Δ) in selected parameters in the placebo and BBR groups.

Parameter	Placebo ΔVEGF (*r*)	Placebo ΔPWV (*r*)	BBR ΔVEGF (*r*)	BBR ΔPWV (*r*)
ΔFat mass corp [kg]	−0.34	−0.29	0.18	−0.19
ΔFLI	−0.30	−0.09	**−0.42**	0.09
ΔhsCRP	**−0.43**	0.18	−0.01	−0.07
ΔBrachial SBP [mmHg]	0.06	−0.18	−0.28	**0.36**
ΔBrachial DBP [mmHg]	0.14	−0.09	−0.20	**0.51**
ΔNumber of waveforms	−0.07	0.03	0.05	0.00
ΔPWA-SP [mmHg]	−0.08	−0.33	−0.24	**0.42**
ΔPWA-DP [mmHg]	0.09	−0.23	−0.20	**0.50**
ΔMAP	−0.13	−0.23	−0.17	**0.51**
ΔPP	−0.16	−0.22	−0.08	−0.16
ΔAp	−0.24	−0.11	0.10	−0.23
ΔAIx	−0.16	0.02	0.28	−0.21

Abbreviations: Δ: change in the parameter; *r*: Spearman correlation index. VEGF—Vascular Endothelial Growth Factor [pg/mL]; PWV—Pulse Wave Velocity [m/s]; PWA-SP—Pulse Wave Analysis Systolic Pressure [mmHg]; PWA-DP—Pulse Wave Analysis Diastolic Pressure [mmHg]; brachial SBP—Brachial Systolic Blood Pressure [mmHg]; brachial DBP—Brachial Diastolic Blood Pressure [mmHg]; MAP—Mean Arterial Pressure, PP—Pulse Pressure; AP—Aortic Pressure; AIx—Augmentation Index; hsCRP—high-sensitivity C-reactive protein; FLI—Fatty Liver Index. Bold text indicates statistical significance: *p*-value < 0.05.

## Data Availability

The original contributions presented in this study are included in the article. Further inquiries can be directed to the corresponding author.

## Abbreviations

The following abbreviations are used in this manuscript:

AIx	Augmentation Index
AMPK	AMP-Activated Protein Kinase
AP	Aortic Pressure
BBR	Berberine
BECs	Biliary Epithelial Cells
BMI	Body Mass Index
BSE	Bovine Spongiform Encephalopathy
CAS	Chemical Abstracts Service
CD34+	Cluster of Differentiation 34 Positive Cells
CFU	Colony-Forming Unit
DBP	Diastolic Blood Pressure
FFQ-6	Food Frequency Questionnaire
FIB-4	Fibrosis-4 Index for Liver Fibrosis
FLI	Fatty Liver Index
GFR	Glomerular Filtration Rate
GMO	Genetically Modified Organism
HRT	Hormone Replacement Therapy
HSC	Hematopoietic Stem Cell
hsCRP	high sensitivity C-Reactive Protein
ICAM-1	Intercellular Adhesion Molecule-1
IDF	International Diabetes Federation
IBD	Inflammatory Bowel Disease
IBS	Irritable Bowel Syndrome
IPAQ	International Physical Activity Questionnaire
MAP	Mean Arterial Pressure
MAFLD	Metabolically Associated Fatty Liver Disease
MAPK	Mitogen-Activated Protein Kinase
MCP-1	Monocyte Chemoattractant Protein-1
MASH	Metabolic Dysfunction-Associated Steatohepatitis
mmHg	Millimeters of Mercury
m/s	Meters per Second
NAFLD	Non-Alcoholic Fatty Liver Disease
NF-κB	Nuclear Factor Kappa-Light-Chain-Enhancer of Activated B Cells
NASH	Non-Alcoholic Steatohepatitis
NO	Nitric Oxide
NSAIDs	Nonsteroidal Anti-Inflammatory Drugs
p	p-Value
PPARγ	Peroxisome Proliferator-Activated Receptor Gamma
PP	Pulse Pressure
PI3K/Akt	Phosphoinositide 3-kinase/Protein kinase B
PWA	Pulse Wave Analysis
PWA-DP	Pulse Wave Analysis–Diastolic Pressure
PWA-SP	Pulse Wave Analysis–Systolic Pressure
PWV	Pulse Wave Velocity
r	Spearman Correlation Index
ROS	Reactive Oxygen Species
SBP	Systolic Blood Pressure
SD	Standard Deviation
SIRT1	Sirtuin 1
Stem Enhance	Stem Cell Mobilizer Supplement derived from Aphanizomenon flos-aquae
T2DM	Type 2 Diabetes Mellitus
TSE	Transmissible Spongiform Encephalopathy
VCAM-1	Vascular Cell Adhesion Molecule-1
VEGF	Vascular Endothelial Growth Factor
VEGF-R1	Vascular Endothelial Growth Factor Receptor 1
VEGF-R2	Vascular Endothelial Growth Factor Receptor 2
VEGF-R3	Vascular Endothelial Growth Factor Receptor 3
WC	Waist Circumference
